# Survey on Security Threats in Agricultural IoT and Smart Farming

**DOI:** 10.3390/s20226458

**Published:** 2020-11-12

**Authors:** Konstantinos Demestichas, Nikolaos Peppes, Theodoros Alexakis

**Affiliations:** Institute of Communication and Computer Systems, Zografou, 15773 Athens, Greece; npeppes@cn.ntua.gr (N.P.); talexakis@cn.ntua.gr (T.A.)

**Keywords:** agriculture, IoT, cybersecurity, threats, security, precision agriculture, smart farming

## Abstract

The agriculture sector has held a major role in human societies across the planet throughout history. The rapid evolution in Information and Communication Technologies (ICT) strongly affects the structure and the procedures of modern agriculture. Despite the advantages gained from this evolution, there are several existing as well as emerging security threats that can severely impact the agricultural domain. The present paper provides an overview of the main existing and potential threats for agriculture. Initially, the paper presents an overview of the evolution of ICT solutions and how these may be utilized and affect the agriculture sector. It then conducts an extensive literature review on the use of ICT in agriculture, as well as on the associated emerging threats and vulnerabilities. The authors highlight the main ICT innovations, techniques, benefits, threats and mitigation measures by studying the literature on them and by providing a concise discussion on the possible impacts these could have on the agri-sector.

## 1. Introduction

According to the latest research results of the United Nations’ Food and Agriculture organization [1], the world will be in need of producing 70% more food in 2050, in comparison with today’s production, in order to feed the constantly growing Earth population, estimated to reach almost 10 billion in 2050 [2]. The market size of the smart agriculture sector is also expected to significantly grow in order to address these needs, supported by the anticipated increase in the number of Internet of Things (IoT) devices employed for agricultural purposes [3].

The continuous and rapid digitization at a global level, underpinned by the progress made in Information and Communication Technologies (ICT), is a trend that deeply transforms many market sectors and has created opportunities in several areas of global economies and societies [4]. The agricultural domain has demonstrated a rapid technological growth in recent years by engaging a variety of emerging ICT solutions. Although Agriculture 4.0 is expected to be the new norm, physical threats and risks in this particular domain is a critical factor that may impede their wide-spread acceptability and adoption. Some of these threats remain traditionally the same, throughout the years, such as weather conditions, but others are associated with the vast evolution of the technological solutions. A brief and accurate list of the physical threats in agriculture is provided by Calicioglu et al. [5], whose work assumes the following classifications: (i) climate change impacts and extreme weather effects; (ii) rapid growth of world population, urbanization and aging; (iii) advanced food production systems and impacts in farmlands and farmers; (iv) pests and diseases.

The most prominent of the aforementioned physical threats for the agriculture sector throughout the years has been the weather conditions, especially in recent decades due to climate change. In this direction, there are several studies aiming to research and elaborate on this type of threat. According to Devendra [6], the problem of climate change is global and affects both developed and developing countries. The authors of this work distinguish the following main effects of the climate change: (i) Earth will become warmer; (ii) soil moisture will decline because of increased temperatures; and (iii) sea level will continue to rise because of the frost melt down. However, although climate change is a major threat for the agricultural sector, agriculture is responsible for part of the problem too. Horrigan et al. claim that about 20% of green-house gases originate from agriculture activities [7].

The physical threats will always be one of major and unpredictable risks in agriculture but there is an upward trend in the recent decades to attempt to minimize their impact by engaging new technologies. To this end, the agricultural domain applications are taking advantage of robust and trustworthy connectivity of different types of equipment, Internet of Things (IoT) networks, and cloud computing infrastructures. For example, the development of smart and precision agriculture applications in order to reduce the existing risks and maximize the production efficiency. Those innovations are only some of the notable achievements that have come as a result of the huge growth of ICT capabilities. On the other hand, with the rapid development of ICT, significant threats of different forms that attempt to exploit security-related vulnerabilities arise as well.

Particularly, the rapid development and adoption pace of IoT has created not only many technological and market opportunities, but also a significant gap and attack surface in terms of security. Despite the fact that many research efforts have concentrated on designing network security protocols, cryptography solutions and device security, several challenges still remain, especially with respect to data integrity, service trustworthiness and the lack of metrics for device security [4]. These challenges are often hard to deal with effectively, due to their range of possible cyber and physical security threats.

Nowadays, ICT systems are the number one cyber-criminal means worldwide for stealing money, intellectual properties, business secrets and other assets. The associated term of cyber-security refers to the combination of techniques, skills and processes in order to ensure a high degree of protection for network, computers, programs and data, against malware, attacks, damage and unauthorized access. In this context, on a daily basis, new types of cyber threats are emerging, including ransomware, endpoint attacks, phishing, third party attacks, supply chain attacks, artificial intelligence and Machine Learning-driven attacks, crypto-jacking, cyber physical attacks, state sponsored attacks, IoT attacks, threats to smart devices, attacks on connected, semi-autonomous or autonomous, vehicles [8]. The significant increase in terrorism, financial devastation and physical injuries or deaths are the result of these types of cyber-attacks. The aforementioned types of threats, alongside the existing physical hazards in agriculture [9], form the basis for creating models that can be used to detect system and network vulnerabilities. The application of cyber tools and (cyber) physical infrastructures as well as of new stronger cybersecurity measures and techniques are of the utmost importance in order to encounter these attacks successfully.

This paper is an extensive literature review of the benefits, the security issues and threats as well as theeffective mitigation strategies posed by the introduction of modern ICT solutions in the domain of agriculture. Advantages, security issues, cybersecurity and IoT-related threats as well as mitigation measures are organized and presented into distinct sections. The remainder of this paper is structured as follows: Section 2 presents an overview of ICT applications and benefits in the agricultural domain; Section 3 focuses on the vulnerabilities, risks and threats in Agriculture 4.0; Section 4 focuses on mitigation strategies and techniques concerning the risks presented in the previous section and finally, Section 5 concludes the paper.

## 2. State-of-the-Art ICT in Agriculture: Definitions, Technologies, Applications and Benefits

Over the last years, the role of ICT and IoT in agriculture is increasingly important [3]. ICT and IoT consists the backbone of the fourth industrial revolution [10], contributing into sustainable growth and development, especially in the agricultural sector of developing countries [11]. The demand for greatly increased productivity and efficiency, at a global level, in conjunction with the need to decrease costs and increase the occupation time, from the employers’ point of view, render these technologies an attractive choice for farmers and companies in the agricultural domain. The integration of IoT into agriculture has resulted in a number of notable applications [12], such as the analysis of crop productivity, crop health monitoring, soil nutrition management, rainfall monitoring, water management and pest infestation monitoring [3]. Systems and IoT applications, such as decision support tools, automated irrigation, frost protection, remote monitoring and fertilization systems, are some typical examples of IoT-based tools that can be deployed and used in the agricultural sector [12,13].

This new form of agriculture which engages ICT and IoT technologies is often denoted as Precision Farming (PF) or Precision Agriculture (PA). Precision Farming or Precision Agriculture, according to Wolf and Wood, is a technological innovation which takes full advantage of the Global Positioning System (GPS) and digitalization of agriculture measurements so as to enhance the accuracy and efficiency of crop production in terms of fertilizer, pest and water management [14]. From this early definition of PA in 1997, the technology evolved rapidly together with the concept of PA itself. Nowadays, Precision Agriculture can be applied and presented also as satellite farming or site-specific crop management (SSCM). PA or SSCM is a farming management concept based on gathering measurements, analyzing and responding to inter- and intra-field variability in crops or livestock [15].

The goal of PA-related research is to design a decision support system (DSS) for the entire farm management, capable to maximize the outcomes by minimizing the inputs and preserving resources [16,17,18]. PA engages a plethora of IoT and electronic sensors for monitoring and controlling the production. Furthermore, until a few years ago, the agricultural domain was typically considered as a heavily mechanized, offline industrial sector. The PA concept came to change that, as there is an increasing trend to move agriculture online. More and more crop and livestock farms interconnect their systems, making them remotely accessible, so that they can easily be monitored and controlled [19]. As already highlighted, PA adopts digital technologies to achieve its purposes [20]. According to the European Commission, object identification, georeferencing, measurement of physical and chemical parameters, satellite navigation, connectivity, data storage and analysis, process automation and vehicle driving are currently the most adopted technologies of PA [20,21].

Thanks to IoT, farmers can get access to collected data in real time and without human intervention. Due to the evolution of technology, the size and shape of sensors is getting smaller and more sophisticated, while in parallel the general cost of the IoT devices is getting lower. The combination of daily-life tools, such as smart phones and/or laptops, with IoT technologies (spanning sensors, gateways, network and middleware devices, as well as additional components for various purposes, e.g., low altitude air-borne hyperspectral imaging system) [22] lead the innovation in the agricultural sector and establish a new domain of interest, often referred to as “Smart Farming”. The ambition of the aforementioned innovations is to confront the industry problems that have occurred over recent decades since the first agriculture revolution in 1970 [3,11].

Some well-known areas where the integration and usage of IoT in agriculture has been applied, are shown in Table 1:

Based on the above applications and analysis of IoT and ICT in agriculture, there are several benefits from the integration of IoT in the agricultural sector. Thus, according to [12,13,54,55,56,57], the benefits from IoT and ICT integration in the agri-sector can be summarized as follows:

- Crop monitoring, decreasing the overall costs;
- Logistic and qualitative traceability of food production combining decision making processes with real-time data for reducing the waste of inputs and overall costs;
- Capitalizing on Big Data resources, at hardware and software level, establishing new agriculture communities in urban and rural areas;
- Generating novel business models in the sector, creating a new retailer–consumer relationship;
- Automatic irrigation systems development that adjust their operations based on the humidity, temperature and soil moisture values which are retrieved through the embedded sensors;
- Collection of environmental parameters, in an automatic way using IoT, which are usable for further analysis;
- Big Data analytic processes and tools for enhancing productivity using decision support systems.

New standards, in combination with the rapid technological advance, lead the agricultural sector, as well as many others, in a new promising future [58]. For agriculture, the main focus of this evolution is the development and innovation of processes that use fewer resources with better production results, keeping in mind that food production is of significant importance for the entire human population.

As digital transformation progresses in the agricultural industry, many businesses face new emerging challenges posed by cyber criminals, who are looking for sectors and organizations that are digitally exposed and might not have built adequate security systems and defensive mechanisms [59]. With the agricultural sector getting more and more dependent on ICT systems, new emerging vulnerabilities, threats and risks, which are described in more detail in the next section, also arise.

## 3. Security Threats in Modern Agriculture

The rapid evolution and the use of smart communication technologies [60] as well as the integration of IoT in addition to the digitization and automation of business, bring new risks and dangers in terms of ICT security in the global market [61]. Potential attacks in various different smart agricultural systems can lead to serious security issues in the dynamic and distributed cyber-physical environment [60,62]. These types of threats and attacks can result in severe disruptions of interconnected businesses. Furthermore, in the heavily mechanized landscape of agriculture, smart technologies and remote administration used in PA and smart farming are something brand new for its stakeholders, with most of the new threats in this specific domain being strongly connected with similar threats that exist in other industries [63]. These threats are mostly related to cybersecurity, data integrity and data loss [64,65]. In addition, because of the fact that the PA sector utilizes heavy machinery connected online, there are many emerging vulnerabilities that can potentially lead to disastrous consequences [65].

The next paragraphs of this section offer a detailed approach on the security threats met in modern agriculture. The extensive literature review performed in this section is focused on the cybersecurity and IoT-related threats in agriculture, which are nonetheless interwoven strongly with PA, Agriculture 4.0 and smart farming [64].

### 3.1. Equipment and Data Vulnerabilities, Risks and Threats in Modern Agriculture

As discussed earlier, the agricultural sector is directly influenced by harsh environmental conditions, such as high temperatures, humidity, rain, winds and other phenomena, which can cause serious damage to electromechanical equipment [66]. All these sensors are susceptible to malfunction, making it possible to provide false measurements and commands which may lead to production disaster [67]. In addition to monitoring and controlling sensors, the wireless networks used in the agri-sector, which are mainly low power such as LoRaWAN, Zigbee, etc., can be affected by the harsh environmental conditions, such as temperature, humidity, obstacles and human presence as well, an impact that leads in turn to communication and data loss [68,69,70]. Moreover, sensors as well as network devices in many cases are physically accessible. This consists a major risk for farmlands, since anyone with malicious intentions can access them either to damage or compromise them in order to make them malfunction [71].

The collected data from IoT sensors and other machinery in agriculture are now transferred online, which constitutes another potential security risk as well. Data privacy and ownership are a major security issue within the agricultural sector [72], as farmers can suffer serious damage, both in financial and personal terms, in the case of a data breach. The threats to confidentiality are classified into four discrete categories in [65], namely: (i) intentional data theft through smart applications and platforms that are not compliant with confidentiality standards; (ii) internal data thefts from a stakeholder in the supply chain in order to damage an agri-business or a farmer; (iii) unethical data sale to minimize profits for farmers or to damage them; (iv) unattended foreign access to sensitive and confidential data through equipment such as drones, sensors, cameras, in order to use them against farmers or to compromise public security [64,73].

In the light of the above, since PA is mainly focused on automated data collection, analysis and decision-making, it is of utmost importance to ensure the integrity and availability of data [64,65,67,74]. Falsification of data, either intentional or unintentional, can have severe consequences, especially since this can affect the decisions made by artificial intelligence (e.g., Machine Learning) algorithms and automation systems leading to the disruption or even the destruction of production [75]. Additionally, false data could lead to dangerous conditions both for the agri-products and human health, possibly resulting in the overuse of fertilizers or pesticides which may travel from the farm to the plate [65].

Alongside the integrity issue, the availability of data and equipment holds a major role in modern agriculture. An elaborate survey for PA in Australia [76] concluded that the majority of farmers make use of GPS systems and are, thus, susceptible to malfunctions that can lead to missing routes for autonomous vehicles and drones and, consequently, to problems in the cultivation procedure [76]. Through this example, it is clear that, in time-precise sectors such as agriculture, the availability of the equipment and systems utilized by farmers is critical [64,65,77].

The aforementioned risks, vulnerabilities and threats exist in every agriculture application which engages modern ICT and IoT technologies. Complementing the aforementioned threats, the next subsection is mainly focused on cybercrime activities and exploitable vulnerabilities in ICT technologies and provides a more detailed and elaborate presentation of the dangers and their causes in terms of cybersecurity in the agri-sector.

### 3.2. Cybercrime and Cybersecurity in Agriculture

Cybercrime is defined as a crime where a computer or a smart device is the object of the crime and/or is used as a tool to commit an offense. A cybercriminal may use a device to access a user’s personal information, confidential business information, government information, or disable a device [78]. Selling or eliciting the abovementioned information online is also considered as a cybercrime. Currently, cybercrime is a worldwide criminal activity and the persons (attackers) that get involved are highly motivated to acquire new technologies, discover and identify new or already existing vulnerabilities and breaches, in order to reach their target and perform an unethical action [60,79,80].

An enormous amount of heterogeneous types of data is transferred through the networks using connected smart devices. The increasing accessibility to smart technology also means that there are multiple access points within users’ homes, offices and any other kind of application or space for hackers to exploit. While law enforcement attempts to tackle this growing issue, crime numbers continue to grow, taking advantage of the anonymity of the internet [78]. Hackers can gain unauthorized access to these types of devices by discovering vulnerabilities at many different layers of ICT systems, such as middleware, network or application layer. It is important for agricultural stakeholders to recognize and protect the ICT environment, as well as to ensure adherence to the current privacy regulations, taking into account the inherent risks of ICT and IoT systems [80].

As an answer to cybercrime, cybersecurity has been an ever-evolving area in recent decades and it will continue to be such, as our world shifts to become more and more online. Individual organizations are highly dependent on supply chains and networks [81], which makes it extremely difficult to manage risks due to the fact that every type of defense is only as strong as its weakest link. Additionally, the existence of one or more possible weaknesses in a third-party application could also put the entire adopted system at risk [82]. Furthermore, even larger firms or companies may have difficulty to recruit the necessary experts, in order to design and develop an adequate defense system against cyber threats and attacks. The larger an organization is, the greater the possibilities are to find weaknesses somewhere in the supply chain [81]. Such weaknesses can provide an access point for cyber criminals, enabling them to launch illegal, devastating attacks on critical infrastructures, such as water or power systems [82,83,84,85].

Nowadays, a cyber-attack to an agricultural or food company is more feasible as the digitization and the use of many devices that are connected to the internet, offer more opportunities to the potential (cyber)criminal in a domain that previously was too difficult to strike or too distant to physically approach [61]. Hence, a possible cyber-attack or security issue in an agricultural company could result in significant human or financial consequences. This suggests that relevant security challenges, arising from the massive and daily use of IoT technology as well as the rise of agroterrorism, demand sufficient attention, security planning and counter measures [61].

Many types of cyber-attacks can cause significant financial and security implications in the agricultural sector, as the majority of system operations are network-based, and on many occasions may not be secured from cyber-threats [86]. Complex combinations of network systems, and especially the dependencies of such systems, amplify vulnerabilities to possible threats by creating possibilities for various cyber threats and causing a cascading effect to materialize. According to Jahn et al. there are five main factors that strengthen these risks in the agriculture sector: (i) the increasing farm consolidation and the associated heavy reliance on technology; (ii) the vertical integration across the food supply chains, where producers may directly trade products and execute processes; (iii) the absence of compliance with food safety, traceability and insurance requirements and regulations; (iv) the increasing dependence of components among food-related systems in smart markets, which leads to greater exposure to failures and faults and (v) the lack of systematic surveillance of food-related systems, social media and markets in a secured, dynamic and near real-time manner, in order to detect significant digital and security issues, which might be the cause of important information breaches and system flaws [87].

This increased risk of cyber-attacks can lead to various ways through which these attacks can impact the agricultural domain. Duncan et al. in [84] classify these ways as: (i) disruption of delivery; (ii) interception of confidential information; (iii) alteration of formulations; and (iv) tampering threats. Obviously, these threats are strongly attached to the adoption and the usage of technological advances in agriculture and food industries. Bogaardt et al. as well as Cooper et al. recognize the main technological advancement areas which are widely adopted in the agri-sector and consist susceptible points for malicious cyber-attacks as follows [86,88]:

- Wireless connections (e.g., Wi-Fi);
- Radio Frequency Identification (RFID);
- Air, soil and crop and infrastructure sensors;
- Unmanned Aerial Vehicles (UAVs), e.g., drones;
- Automation systems focused on PA (e.g., Real Time Kinematic Technology);
- Mobile devices (e.g., laptops, mobile phones, GPS trackers);
- Vertical farming and smart agriculture;
- The combination of biotechnology and nanotechnology with Artificial Intelligence (AI).

The agricultural sector is mainly interwoven with people’s nutrition so every risk, vulnerability and threat represents a major risk for public health. Cybersecurity is, in general, an open and evolving research field and when it comes to the agricultural domain and its application, its significance is of utmost importance. The survey conducted for cybersecurity in agriculture and its threats can be summarized in Table 2.

### 3.3. IoT Vulnerabilities, Risks and Threats in Agriculture

The main cybersecurity threats were extensively presented in the previous subsection. Every and each of these threats are also applicable to IoT technologies and consist a threat for the associated agricultural applications. In the following paragraphs, a more detailed and focused survey on the IoT domain and its threats in agriculture is presented. IoT technologies are widely adopted by agricultural stakeholders in every part of the world and in every phase from production to supply chain. Thus, their role and their susceptibilities and risk are of high importance and worthy of study.

IoT technology is consisted of four major systems, which render the communication between two endpoints (nodes) feasible. An IoT system technology is composed of: (i) the sensing technology; (ii) IoT gateways; (iii) cloud server/data storage and (iv) remote control using mobile applications.

Current along with new risks and vulnerabilities come along with new as well as older types and architectures of IoT systems in agriculture. Such vulnerabilities can be attributed to hardware and software issues of the IoT devices, communication protocols, as well as data storage and processing solutions (e.g., in cloud infrastructures, data centers and smartphones) [2,11]. A brief but accurate categorization of the main causes of low security in IoT include, according to [2,104,105], the following:

- Unpatched firmware and/or extended use of default passwords, which allow for compromising the devices within an IoT network;
- Limited computational resources of smart devices, which hinders the implementation of complex cryptographic algorithms, due to the efforts of vendors to reduce the costs of their products in a competitive environment;
- Vulnerabilities in the communication protocols used by smart devices (e.g., ZigBee, Bluetooth);
- Low security level of the old version of the Wi-Fi Protected Access (WPA) protocol, which is still used in many cases;
- Search engines that can be used for executing passive vulnerability detection;
- The danger of organizing millions of smart devices in a powerful botnet (e.g., Mirai), due to the relatively easy detection of vulnerabilities by means of internet scanning;
- General lack of attention to the security of smart devices.

Concisely, the overall lack of security of smart devices is caused by the general absence of standards or mandatory official recommendations for the security of IoT. The lack of legislative acts that regulate the responsibility between the manufacturer, the seller and the client, as well as the existence of a diversity of devices, combined with the need for costs savings, jointly affect the security of IoT [2,78].

Finally, IoT vulnerabilities, risks and threats can be classified using several different criteria. These threats apply to IoT applications in general, also including the agricultural domain. Firstly, there are two main classification categories of security threats in IoT applications in the agricultural sector, namely: (i) internal vs. external threats; (ii) passive vs. active threats [79]. Another interesting classification of threats for IoT follows a layer-based approach. Hassija et al. [22] propose that each of the following layers combines diverse technologies that bring a number of possible vulnerability issues, breaches and/or security threats. The architecture of IoT depends upon five distinct layers: (i) the application layer; (ii) the middleware layer; (iii) the internet layer, (iv) the access gateway layer and (v) the edge technology layer. Taking into account the layered architecture of a typical IoT system and the diverse technologies that it adopts, there is a significant number of possible vulnerability issues, security breaches and threats, which can result in major issues and/or problems for the organizations into the agriculture domain. Possible attacks on each layer of a typical IoT system are classified as depicted in Table 3:

## 4. Mitigation Measures and Strategies for Security Threats in Agriculture

In the previous section, the main vulnerabilities, risks and threats in agriculture concerning the adoption and utilization of state-of-the-art technology, techniques and tools were discussed. Thus, it is of high importance for agriculture stakeholders to invest and adopt measures and strategies which can mitigate the risks and avoid disastrous consequences. Nevertheless, despite the increasing concern related to cyber agroterrorism, a large number of companies are still not investing sufficiently in the improvement of the cybersecurity protection of IoT and ICT systems [61]. Although, many larger corporations and industries have invested in safe and efficient security systems and measures, smaller agricultural companies and farms often lack in terms of financial resources, time and plans to design and implement adequate measures and/or systems against possible cyber-attacks [83].

Agriculture stakeholders must focus on the possible attacks featured in detail previously such as: data privacy and integration, risk management (also in relation to third parties), integrity protection, non-repudiation, trusted origin, access control, scalability and robustness in terms of points of failure [62,79,80,112] in order to enhance cybersecurity. To address these challenges, some emerging architectures embedded blockchain technology in order to ensure data privacy and integrity, addressing space, fault tolerance, trusted origin and accountability, access control, removal of third-party risks, prevention of illegal use of personal (private) data, as well as protection of the sharing of IoT network information [112,113]. In summary, prior research for the enhancement of security and privacy in IoT and ICT applications in the agricultural sector falls under six main categories according to Ferrag et al. [79]: (i) privacy-preserving solutions; (ii) data integrity solutions; (iii) authentication solutions; (iv) access control solutions; (v) data confidentially solutions; and (vi) blockchain-based solutions and consensus algorithms.

Cybersecurity and mitigation measures and strategies is a very wide, open and rapidly evolving research field. Every day new threats and risks are emerging and thus new countermeasures become available. Table 4 features a list of countermeasures that can be taken against security aspect threats and IoT layer threats as those described in Table 2 and Table 3.

Lastly, regarding agricultural cyber-security, researchers intensify their efforts in designing Machine Learning (ML) and Deep Learning (DL) methods for anomaly behavior detection and network analysis of intrusion detection systems (IDS) [124]. Traditional Machine and Deep Learning applications applied for cybersecurity in agriculture include [125,126]:

- Support Vector Machine (SVM);
- K-Nearest Neighbor;
- Decision Trees;
- Deep Belief Network (DBN);
- Recurrent Neural Networks (RNN);
- Convolutional Neural Networks (CNN);
- Artificial Neural Networks (ANN);
- Self-Organizing Maps (SOMs);
- Natural Language Processing (NLP);
- Biologically inspired techniques, such as Deep Neural Networks (DNN) and/or Generative Adversarial Networks (GANs).

The emergence of such methods has enabled efficient handling and analysis of large amounts of data (e.g., files extracted from server logs, communication equipment, security solutions and blogs related to information security, in different types of structured and unstructured formats), so as to support a variety of conceptual models, security activities and knowledge generation mechanisms. This leads to efficient decision making or automation of security responses without the need for human interception [127].

## 5. Conclusions

The goal of this study was to conduct a thorough literature research on the benefits and the security threats imposed by the introduction of novel ICT technologies in the agricultural sector nowadays as well as on the associated mitigation measures and strategies. Research began with the advantages of ICT technologies and their importance in the agri-sector and then focused on the threats emerging alongside the evolution of smart agriculture or Agriculture 4.0. Also, research conducted in the context of this paper showed that, despite the rapid evolution in technology, stakeholders throughout the entire agricultural sector need to exercise caution on how they adopt and exploit new technologies and tools. The agri-sector tends to be even more vulnerable compared to other sectors that engage digital tools, so the mitigation of security threats is and will be an ever-open research subject.

The new era in agriculture employs concepts and assets from Industry 4.0 that facilitate the transition to Agriculture 4.0 and the smart farming era. The increasing complexity of methods used for cultivation and production of agri-products leads to even more potential threats on various facets. By making the agriculture ecosystem smart and connected, multiple “backdoors” have opened with stakeholders and security specialists urged work together to block them entirely, in order to guarantee the safety and efficiency of all the new possibilities offered to farmers.

To conclude, in order to characterize a new method or system as successful, it should be able to: (i) reduce costs; (ii) save time; (iii) increase trust; (iv) reduce risk. Stakeholders in agriculture should be willing to adopt new ways of working only when they are convinced that the newly proposed method or system is secure, safe and usable, increases productivity and brings added value. Taking the above into account, it becomes clear that the consolidation of new technologies in the sensitive sector of agriculture is an enormous challenge which should be carried out step by step, and only by efficiently engaging the directly affected stakeholders across the supply chain in security-preserving activities and investments.

## Figures and Tables

**Table 1:** Overview of Internet of Things (IoT) usage and integration areas and corresponding studies.

Area	Studies
Continuous land monitoring	[3,23,24,25,26,27]
Water management	[1,28,29,30]
Monitoring and reporting of crop growth	[25,31,32,33,34,35]
Identification and management of soil characteristics	[36,37,38,39,40]
Detection and recognition of diseases in crops and/or plants	[41,42,43,44,45]
Enhanced food preservation and quality control	[46,47,48,49]
Smart livestock	[50,51,52,53]

**Table 2:** Overview of possible attacks per security aspect in agricultural cybersecurity.

Security Aspect	Examples of Attacks	Agriculture Consequences	Studies
Privacy	Physical Attack Replay Attack Masquerade Attack	The collection of information regarding the type and possible usage of devices concerning agriculture projects. These security leaks can be used in order to get access to infrastructure and production standards as well as getting privacy data and compromising the privacy of the system. Theft and vandalism purposes can be the outcome of a possible violation of privacy.	[62,78,79,89,90,91,92]
Confidentiality	Tracing Attack Brute Force Attack Known-Key Attack	The usage of various communication devices in a smart farming or an agriculture system based on ICT can outcome into data travelling through several interconnected devices and protocols from source to destination. Possible confidentiality problems can lead to the persistence, on many occasions, of loss of privacy and data or information breaches. The unauthorized access to important data as a result of the confidentiality loss could lead to theft of key information and also cause serious threats over the involved agriculture system users’ confidential information.	[29,62,79,93,94]
Integrity	Forgery Attack Man-In-The-Middle Attack (MITM) Biometric Template Attack Trojan Horse Attack	As a result of possible unauthorized or improper changes in the trustworthiness of data or resources, information between agriculture ICT or smart farming systems can be no longer reliable or accurate. The transmitted information data between the devices and/or the people/farmers/stakeholders that are involved in an agriculture business or even a process can lead to possible financial or authentication frauds due to the lack of the assurance that the information is sufficiently accurate for its purpose.	[62,79,91,95,96]
Availability	Denial of Service (DoS) attacks (SYN Flood, Ping of Death, Botnets)	A smart farming environment is meant for real or near-real time operations in order to keep a real-world impact. An attacker can suspend the activities of the installed smart farming network or even establish the services unavailable to the farmers. The lack of availability of the provided services can lead to business disruption, possible loss of customer’s confidence and revenue.	[62,79,92,97,98]
Authenticity	Attacks against Authentication (Dictionary attack, Session Hijacking, Spoofing)	Authenticity ensures the authentication of certain information provided from a valid/authorized source. Forged attackers’ identities can mimic legal/authorized persons and gain access to the smart farming system. Possible results can be the data breach/loss and/or alternation, service unavailability, loss of devices connectivity or even smart farming agriculture system corruption and/or destruction.	[62,79,99,100,101]
Non-Repudiation	Malicious Code Attack Repudiation Attack	During the authentication process, a commonly known service that provides proof of the integrity as well as the origin of data, both in an unforgeable relationship, and can be verified by any third party at any time with high assurance and genuineness, is non-repudiation. The repudiation of information allows an attacker to repudiate all the power consumption, generated information and production processes of an agriculture ICT system, which can lead to a situation of refusing services, authentication information or data transmissions, through the nodes of the system.	[62,79,102,103]

**Table 3:** Overview of security threats in agriculture across different IoT layers.

Layer	Security Threats	Smart Farming Effects	Studies
Application	Data Thefts Access Control Attacks Service Interruption Attacks Malicious Code Injection Attacks Sniffing Attacks Reprogram Attacks	The top of the stack in the already mentioned IoT layer architecture. Possible effects or problems could be considered the lack of the delivery of services between the respective users from various domains such as farmers, retailers and/or other stakeholders. Accessibility problems for the involved users and lack of security and privacy are also major issues.	[62,79,106,107,108]
Middleware	Man-In-the Middle Attack (MITM) SQL Injection Attack Signature Wrapping Attack Cloud Malware Injection Flooding Attack in Cloud	This layer operates in two-way mode. More specifically, this layer stands between (in the middle) of the application and the hardware layer and also acts as an interface between them. Major problems that come as a result of attacks on this layer can affect data and/or device (nodes installed into the agriculture infrastructure) management and other types of issues such as device information discovery, access control by the users and data analysis as well.	[62,79,107,109]
Internet	Phishing Site Attack Access Attack DDoS/DoS Attack Data transit Attacks Routing Attacks	The most crucial layer concerning the establishment of the communication between two distinct endpoints, such as device-to-device, device-to-cloud, device-to-gateway and back end data-sharing. In case of a failure the communication is being disrupted and the ICT system is, practically, out of service. Improper communication services and/or lack of automatic updates could lead to privacy concerns among the users’ private information (e.g., access credentials)	[62,79,107,110]
Access Gateway	Secure on-Boarding Extra Interfaces End-to-End Encryption Firmware Updates	Access GW layer contributes to the handling of the very first data as well as to bridging the gap between the client (farmers, stakeholders, retailers) and the end point (node or device). Messages routing, identification and subscribing problems between the smart farming nodes could be possible outcomes to the client side concerning the final form of the received message. This message may also include the desired information as well as lack of transport encryption/integrity verification, so sensitive data could easily then be intercepted.	[62,79,107,108,111]
Edge Technology	Node Capturing Malicious Code Injection attack False data Injection Attack Side-Channel Attacks Eavesdropping and Interference Sleep Deprivation Attacks Booting Attacks	This layer is consisted of the majority of hardware parts (e.g., sensors, Radio-Frequency Identification (RFID) tags) and has a significant role for the communication between the involved devices as well as the data collection within the network and the servers that are deployed on the installed ICT smart farming system. Possible attacks on the entities of this layer could lead to important problems of monitoring or sensing various phenomena. Additionally, information theft and/or tampering could also be possible results.	[62,79,107,108,109]

**Table 4:** Mitigation countermeasures against cybersecurity threats and attacks.

Mitigation Measures	Short Description	Security Aspect Threat	IoT Layer Threat	Studies
Firmware Update	It is important to be checked if an update mechanism is installed or even turned on in the device, in order to prevent various online and offline attacks.	Privacy, Confidentially, Availability	Application	[92,106,107,114,115,116,117,118,119,120]
Block Unnecessary Ports	Block unnecessary, vulnerable or overlooked ports, so to prevent a possible cyberattack or device exploitation.	Privacy, Authenticity, Non-Repudiation	Internet	[100,101,107,110,114,115,116,117,118,120,121]
Disable Telnet	Telnet is a great security risk due the fact of sending passwords and usernames in clear text. It should be ensured that is turned off.	Privacy, Authenticity, Non-Repudiation	Internet	[100,101,107,110,114,115,116,117,118,120,122]
Encrypted Communication Usage (SSL/TLS)	Using a secure protocol such as SSL/TLS consists an essential step towards a device security through transport encryption.	Confidentially, Integrity, Authenticity	Access Gateway	[93,94,108,111,114,115,116,117,119,120]
Strong Passwords	A token could be used to increase the security level of the device.	Privacy, Confidentially, Authenticity	Middleware	[93,94,100,107,109,114,115,116,117,120,123]
Encryption of Drives	Keep the data inaccessible in case of a device theft.	Privacy, Integrity	Middleware, Edge Technology	[93,94,100,101,107,108,109,114,115,116,117,120,123]
Accounts Lockout	Account lockout mechanism(s) should be incorporated in the device so as to allow a legitimate user to access and retrieve information.	Privacy, Confidentially, Authenticity	Edge Technology	[93,94,100,101,107,108,109,114,115,116,117,123]
Periodic Assessment of Devices	Devices need periodic cybersecurity assessments in order to check and avoid possible new vulnerabilities of any type.	Privacy, Availability	Edge Technology	[93,94,100,101,107,108,109,114,115,116,117,120]
Secure Password Recovery	Helps to retrieve back missed credentials in a secure way.	Privacy, Confidentially, Authenticity	Middleware	[93,94,100,107,109,114,115,116,117,118,119,120]
Two-Factor Authentication	Keep data encrypted and protected as well.	Privacy, Confidentially, Authenticity	Middleware	[93,94,100,107,109,114,115,116,117,118,120]
Disable UPnP	In order to avoid possible exposure of the network to aspiring attackers, the UPnP must be disabled as it does not require any authentication, which renders it an important security flaw.	Privacy, Confidentially, Availability	Internet	[93,94,100,107,110,114,115,116,117,120]
